# Targeting XIAP for Promoting Cancer Cell Death—The Story of ARTS and SMAC

**DOI:** 10.3390/cells9030663

**Published:** 2020-03-09

**Authors:** Ruqaia Abbas, Sarit Larisch

**Affiliations:** Laboratory of Cell Death and Cancer Research, Biology& Human Biology Departments, Faculty of Natural Sciences, University of Haifa, Haifa 3498838, Israel; ruqaiaa.92@gmail.com

**Keywords:** XIAP, cIAPs, ARTS, Smac, IAP antagonist, small molecules, apoptosis, cancer therapy

## Abstract

Inhibitors of apoptosis (IAPs) are a family of proteins that regulate cell death and inflammation. XIAP (X-linked IAP) is the only family member that suppresses apoptosis by directly binding to and inhibiting caspases. On the other hand, cIAPs suppress the activation of the extrinsic apoptotic pathway by preventing the formation of pro-apoptotic signaling complexes. IAPs are negatively regulated by IAP-antagonist proteins such as Smac/Diablo and ARTS. ARTS can promote apoptosis by binding and degrading XIAP via the ubiquitin proteasome-system (UPS). Smac can induce the degradation of cIAPs but not XIAP. Many types of cancer overexpress IAPs, thus enabling tumor cells to evade apoptosis. Therefore, IAPs, and in particular XIAP, have become attractive targets for cancer therapy. In this review, we describe the differences in the mechanisms of action between Smac and ARTS, and we summarize efforts to develop cancer therapies based on mimicking Smac and ARTS. Several Smac-mimetic small molecules are currently under evaluation in clinical trials. Initial efforts to develop ARTS-mimetics resulted in a novel class of compounds, which bind and degrade XIAP but not cIAPs. Smac-mimetics can target tumors with high levels of cIAPs, whereas ARTS-mimetics are expected to be effective for cancers with high levels of XIAP.

## 1. Introduction

Apoptosis is a form of programmed cell death that is critical for normal development and tissue homeostasis. Abnormal regulation of this process is associated with a wide variety of human diseases, including immunological and developmental disorders, neurodegeneration, and cancer [1,2,3]. Apoptosis can be initiated by both extrinsic and intrinsic signals mostly centered in and from the mitochondria [4,5]. The extrinsic apoptotic pathway is activated when apoptotic inducing ligands such as Fas ligand (FasL) and tumor necrosis factor alpha (TNFα) engage with their receptors, such as FAS receptor (CD95) and TNF receptor (TNFR), respectively [6]. The intrinsic pathway is induced by internal apoptotic signals (such as DNA damage) but can also be activated following extrinsic stimuli to enhance the death receptor apoptotic signals [7,8]. Both pathways are executed by activating caspases (cysteine–aspartic proteases) through cleavage from their inactive zymogens [9,10,11]. Apoptotic caspases are organized into “initiator caspases” (caspase-2, -8, -9, and -10) and effector caspases (caspase-3, -7, and -6) [11,12,13,14]. Caspases 8 and 10 are cleaved primarily in response to extrinsic signals, while caspase-9 is activated in the intrinsic mitochondrial pathway. These enzymes act in a cascade that culminates in cleavage of multiple cellular proteins, resulting in disassembly of the content of cells [10]. In living cells, caspases are kept in check by inhibitors of apoptosis (IAP) proteins [15,16]. There are eight human IAPs, namely X-linked-IAP (XIAP), cIAP1, cIAP2, ML-IAP, NAIP, ILP2, survivin, and Bruce. IAPs contain between one to three baculoviral IAP repeats (BIR), which serve as protein–protein interaction domains [9,10,17]. In addition, XIAP, cIAP1, cIAP2, ML-IAP, and ILP2 have an ubiquitin-associated (UBA) domain, which enables the binding of poly-ubiquitin conjugates, and a RING domain responsible for E3-ligase activity [18,19,20,21]. The best-studied IAP is XIAP, which has three BIR domains. Its BIR3 domain binds directly to and inhibits caspase-9, while the linker region between the BIR1 and BIR2 domains is responsible for the inhibition of caspase-3 and 7 [22,23,24]. XIAP is the most potent member of the IAP gene family in terms of its ability to directly inhibit caspases and suppress apoptosis [25]. Unlike XIAP, its two paralogs, cIAP1 and cIAP2, are not able to directly repress caspases [15,17]. cIAPs can interact with TNF-associated factors (TRAFs) to prevent the formation of pro-apoptotic signaling complexes in the extrinsic apoptotic pathways initiated by TNFR [26,27,28,29]. cIAPs affect cell survival through both canonical and non-canonical NF-κB signaling [28,30,31,32,33,34,35]. The canonical NF-κB pathway involves the assembly of a signaling complex comprised of TRADD, TRAF2, RIPK1, and cIAPs. cIAPs induce a non-degradative ubiquitylation of RIPK1 as well as auto-ubiquitylation [26,27,28,29]. This leads to activation of downstream pro-survival NF-κB signaling. Alternatively, survival is maintained through the inhibition of the non-canonical pathway by cIAPs promoting proteasomal degradation of NIK (NF-κB-inducing kinase) [26,27,28,29,30,31,32,33,34,35]. Notably, inactivation of individual cIAP genes yields viable mice and causes no obvious defects in NF-κB signaling and TNFα induced cell death, presumably due to redundant function [36,37]. Consistent with this idea, cIAP1/2 DKO mice die as embryos and have a reduced response to TNFα that has been attributed to a defect in the amplification loop of the TNFR pathway [38]. In contrast, although originally XIAP deficient mice were reported to have no obvious phenotype, a later publication found that XIAPΔRING mutant mice develop lymphomas and lymphoblastic leukemia [19,39]. In addition, inactivation of XIAP sensitizes certain types of stem cells for apoptosis, including hair follicle stem cells of the skin and intestinal stem cells, and this is associated with decreased wound healing [40,41]. Moreover, using gene-targeted mice, the loss of XIAP or deletion of its RING domain lead to excessive cell death [42].

## 2. IAP-Antagonists, Smac/Diablo, and ARTS

IAPs are negatively regulated by IAP-antagonist proteins, such as Smac (second mitochondrial-derived activator of caspases)/Diablo (from here forth will be referred to as Smac), Omi/HtrA2, XAF1 (XIAP-associated factor 1), and ARTS (Apoptosis Related protein in the TGF-β Signaling pathway) [43,44,45,46,47,48,49,50]. The name “ARTS” reflects the fact that this protein was originally discovered in cells induced for apoptosis by TGF-beta [47]. Yet, we have found that ARTS acts downstream of basically all apoptosis stimuli tested, such as treatment with STS (staurosporine), etoposide, arabinoside (Ara-c), nocadosole, UV radiation, TNF-α, etc. [47,51,52]. Smac and Omi/HtrA2 contain a conserved four amino acid domain (AVPI/F) that was first described in the *Drosophila* IAP-antagonists reaper, hid, and grim, later termed IBM (IAP-binding motif) [53,54,55,56]. Genetic and biochemical characterization of reaper, hid, grim, and Diap1 (*Drosophila* IAP1) provided the first evidence for the critical physiological role of IAPs and their antagonists in regulating apoptosis [55,57,58,59,60]. In this review, we will concentrate on Smac and ARTS (Table 1), which represent the two major types of IAP-antagonists, with a focus on developing small-molecule mimetics of these IAP-antagonists for cancer therapy. Smac is localized at the inner membrane space of mitochondria [43,44,61]. Upon apoptotic induction and mitochondrial outer membrane permeabilization (MOMP), Smac, and cytochrome C (Cyto c) are released into the cytosol from the mitochondrial inner membrane space. Cyto c together with APAF-1 and pro-caspase-9, then form the "apoptosome" complex which cleaves and activates caspase-9 [62]. Smac binds to the caspase-9 pocket in BIR3 domain of XIAP via its IBM, resulting in the release of XIAP-bound-caspases [43,63,64,65]. Importantly, the release of Smac from the mitochondria is caspase dependent [63,66,67,68]. This indicates that caspases are activated upstream of MOMP, and the release of Smac and Cyto c from mitochondria [67,69]. Smac binds to cIAP1, cIAP2, and XIAP, yet it only induces the ubiquitylation and degradation of cIAPs but not XIAP [70,71]. There are two possible interpretations for the binding of Smac to XIAP. The prevailing theory is that Smac antagonizes XIAP. On the other hand, Smac may be a substrate for XIAP-mediated degradation. Consistent with this idea, it has been reported that XIAP can degrade Smac and thereby attenuate apoptosis [72].

Interestingly, Smac over-expression alone, without any additional apoptotic stimuli, does not cause either apoptosis or induction of caspase activity [43]. Moreover, *Smac* KO mice developed normally and did not exhibit any obvious macroscopic or microscopic abnormalities [73]. Aged mice (more than 12 months of age) did not show any sign of anomalies, such as autoimmune disease or tumor formation [73]. Notably, *Smac* KO cells were resistant to apoptosis induced by NSAIDs and TRAIL, yet treatment with other agents did not significantly affect these cells [74]. Furthermore, loss of *Smac* in mice led to elevated levels of cIAP1 and cIAP2 [74,75]. Yet expression levels of XIAP remained intact in *Smac* KO cells [63] (summarized in Table 1). These data imply that Smac is required for the inhibition of cIAPs but not XIAP in vivo and suggest the existence of a redundant molecule/s capable of compensating for the loss of Smac function [73,74]. 

**Figure 1:**
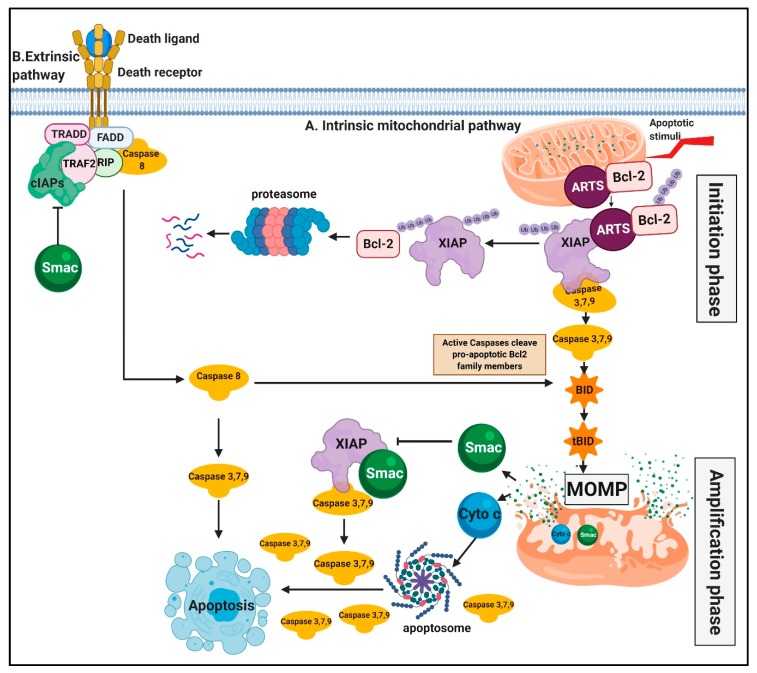
Schematic representation of the role of ARTS and Smac in induction of apoptosis. **A**. Intrinsic mitochondrial pathway: Initiation phase: Upon induction of apoptosis, ARTS binds directly to XIAP and brings it into a ternary complex with Bcl-2. This stimulates ubiquitin-proteasome-mediated degradation of Bcl-2 and XIAP resulting in de-repression of pre-apoptosome active caspases. Amplification phase: According to this model, non-lethal amounts of active caspases, cleave Bid (and possibly other pro-apoptotic Bcl-2 family members) and promote mitochondrial outer membrane permeabilization MOMP). During MOMP, Smac/Diablo (Smac) and cytochrome c (Cyto c) are released from the inner membrane space of the mitochondria into the cytosol. This further stimulates the activation of caspases, and contributes to a cascade of caspase activation-amplification loop. Smac binds to XIAP and promote degradation of cIAPs which results in caspase activation. cIAPs also interact with TRAF and help activate the TNFR signaling pathway. **B**. Extrinsic pathway: Death ligands binding to death receptors cause the activation of caspases and cell death. The extrinsic and intrinsic pathways crosstalk via caspase-induced-cleavage of BID. Truncated Bid (tBID) promotes MOMP and further activation of caspases leading to apoptosis. Figures were generated using biorender.com.

ARTS (Sept4_i2) is a splice variant derived from the Sept4 (Septin 4) gene, and the only splice variant that functions as a pro-apoptotic protein [76]. ARTS is a tumor-suppressor protein that is localized at the mitochondrial outer membrane (MOM) [69]. Upon apoptotic stimuli, ARTS rapidly translocates to the cytosol in a caspase-independent manner and antagonizes XIAP [50,69]. ARTS binds directly to the XIAP/BIR3 domain but in a way distinct from Smac. ARTS does not contain a canonical IBM; instead, it binds to XIAP/BIR3 using unique sequences found at its C-terminus [50,77,78]. Moreover, ARTS binds to specific sequences within XIAP/BIR3, which are not interacting with Smac. Therefore, the binding sites of ARTS and Smac within BIR3/XIAP are proximate but do not overlap [77,79]. Moreover, ARTS also binds to the UBA domain and has contact points in the BIR1 and BIR2 domains of XIAP [80]. Importantly, ARTS is the only IAP-antagonist that can induce degradation of XIAP through the ubiquitin proteasome-system (UPS) [67,69,80]. ARTS promotes the auto-ubiquitylation and degradation of XIAP in addition to serving as an adaptor bringing the E3-ligase Siah to stimulate the degradation of XIAP [80]. Moreover, ARTS acts as a scaffold by bringing XIAP with its E3-ligase activity, into close proximity with Bcl-2, promoting UPS-mediated- degradation of Bcl-2 (Figure 1) [67]. Thus, ARTS functions as a dual antagonist of both XIAP and Bcl-2 to initiate MOMP and apoptosis. Furthermore, the translocation of ARTS from the mitochondrial outer membrane (MOM) to the cytosol precedes MOMP and the release of Cyto c and Smac, and is required for it [67,69]. The localization of ARTS at the MOM, facilitates its rapid translocation to the cytosol and binding to XIAP, minutes following apoptotic stimuli [69]. The direct binding of ARTS to XIAP enables de-repression of caspases which are required for MOMP, and the subsequent release of Cyto c and Smac [63,66,67,68,69,81,82,83,84]. We termed this pre-MOMP stage of releasing active caspases from their inhibition by XIAP, the initiation phase (Figure 1). This initial de-repression of non-lethal active caspases from XIAP can now mediate the cleavage of protein substrates, such as Bid, and possibly other pro-apoptotic Bcl-2 family members, which are known to promote MOMP (Figure 1) [69]. The process of MOMP allows the release of Cyto c and Smac from the inner membrane space of the mitochondria. This will now promote further amplification of caspase activation through formation of the apoptosome complex, and Smac antagonizing IAPs. We termed this stage the amplification stage (Figure 1). ARTS-deficient cells exhibit a significant inhibition in MOMP and delayed release of both Smac and Cyto c [69]. Thus, ARTS acts upstream of mitochondria to initiate caspase activity, which is important for the proper execution of mitochondrial outer-membrane permeabilization (MOMP) (Figure 1).

Over-expression of ARTS alone is sufficient to induce cell death in a variety of cultured cancer cell lines in addition to increasing the susceptibility of cells toward apoptotic inducers [47,52]. Human and mice studies have shown that ARTS functions as a potent tumor suppressor protein. ARTS expression is lost in more than 70% of acute lymphoblastic leukemia (ALL) patients [51], in 50% of lymphoma patients [85], and in a significant fraction of hepatocellular carcinoma (HCC) patients. Studies using *Sept4*/ARTS-null mice showed that ARTS is a physiological antagonist of XIAP in vivo. In particular, *Sept4*/ARTS null mice have increased numbers of hematopoietic stem and progenitor cells (HSPCs), which are resistant to apoptosis [85]. Deletion of *Sept4*/ARTS equips the intestinal stem cells (ISCs) niche with increased resistance against apoptosis [41]. In addition, *Sept4*/ARTS deficient mice have elevated numbers of hair follicle stem cells (HFSCs) that are protected against apoptosis and display marked improvement in wound healing and regeneration of hair follicles [40]. These mice exhibit spontaneous accelerated tumor development and elevated XIAP levels [40,41,85,86]. These data suggest that the pro-apoptotic function of ARTS as an XIAP-antagonist along with its function in stem cells may serve to inhibit the emergence of cancer [86]. Moreover, the resistance of *Sept4*/ARTS-null hematopoietic stem and progenitor cells (HSPCs) to apoptosis and the cell-autonomous lymphoproliferation is suppressed by the loss of XIAP function in *Sept4*/ARTS/XIAP double-knockout mice [75]. Collectively, these results demonstrate the important physiological role of ARTS in regulating apoptosis and tumor suppressor in vivo through its role as a specific XIAP-antagonist (Table 1). A detailed comparison of the features of Smac and ARTS is shown in (Table 1). 

The differences between the two IAP-antagonists, ARTS and Smac, are summarized in Table 1. These data indicate that Smac functions as a more specialized cIAP- antagonist, significantly effecting the TNFα (tumor necrosis factor)/TRAIL pathway, whereas ARTS acts as a physiological XIAP- antagonist.

## 3. Targeting XIAP for Cancer Therapy; Developing Smac and ARTS Small Molecule Mimetics

**Figure 2:**
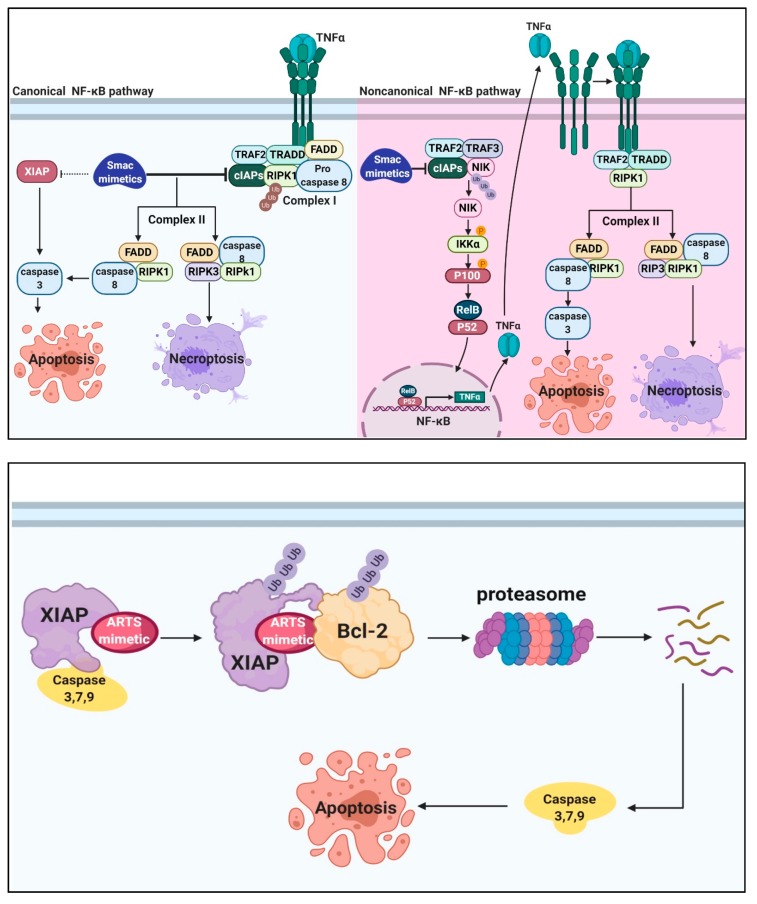
Models for Smac-mimetic (SM) and ARTS-mimetic (AM) mode of action. (**A**) Smac-mimetic (SM) mode of action. Treatment with Smac mimetics inhibits the NF-κB canonical pathway (right) by binding and degrading cIAPs. This prevents the ubiquitylation of RIPK1 (non-degradative, brown Ub) and leads to the formation of a complex containing RIPK1, caspase 8, and FADD, which promotes apoptosis. In addition, Smac mimetics-induced-degradation of cIAPs prevent the degradation of NIK (NF-κB inducing kinase), which in turn stabilizes NIK and activates the non-canonical NF-κB pathway (left). The stabilized NIK phosphorylates IKKα, which in turn phosphorylates p100 and generates the p52 protein. RelB-p52 heterodimers then translocate to the nucleus and activate the expression of NF-kB pro-apoptotic target genes. NF-kB-mediated-induction of TNFα results in activation of the TNF-receptor (TNFR) extrinsic pathway. This activation induces formation of the complex containing RIPK1, caspase-8, and FADD, which promotes apoptosis. Cells expressing high levels of RIPK3 undergo necroptosis. Smac mimetics can also bind XIAP and hence may contribute to de-repression of caspases to induce apoptosis. Figures were generated using biorender.com. (**B**) ARTS-mimetics (AM) mode of action. AM bind XIAP, which may induce an allosteric conformational change resulting in activation of XIAP E3 ligase activity. This leads to auto-ubiquitylation and proteasomal degradation of XIAP. In addition, the XIAP-AM complex can bring XIAP into close proximity with Bcl-2 which allows its ubiquitylation and proteasome-mediated degradation leading to apoptosis. Figures were generated using biorender.com.

Many tumors over-express XIAP and cIAP1, thereby allowing cancer cells to escape apoptosis [3,89,90]. XIAP is overexpressed in leukemia, lung, colon, melanoma, ovarian, bladder, renal, breast, prostate, and thyroid carcinomas [91,92,93]. cIAP1 is over-expressed in colon, bladder carcinomas, and cervical B-cell chronic lymphocytic leukemia [91,93]. Therefore, XIAP and cIAPs have become attractive targets for cancer therapy [45,51,88,94]. Most of the efforts to target IAPs were focused on developing Smac (IBM) mimetics [64,65,95,96,97,98,99]. Here we will review the progress in developing Smac-based IAP-antagonists, and the initial efforts to develop ARTS-based small-molecule mimetics. In recent years, intense efforts were made to target IAPs and in particular XIAP for cancer therapy. Most approaches have focused on derivatives of the IBM tetra-peptide, but anti-sense oligonucleotides (ASO) have been generated as well [97,100]. AEG35156 is an ASO that was designed to bind to XIAP with maximal stability and potency. AEG35156 has an acceptable safety profile with some signs of anti-cancer activity. However, treatment was limited to only two cycles in average due to the appearance of transaminitis (liver toxicity). Furthermore, the combination of AEG35156 with different standard-of-care cytotoxic agents caused a reversible peripheral neuropathy [101]. Therefore, more studies are needed to define appropriate indications and drug combinations for AEG35156 therapy [101,102]. Small-molecule Smac mimetics were based on the conserved IBM (AVPI/F) of natural IAP-antagonists that is found in reaper, hid and grim, Smac and Omi [54,55,57,58,64,95,96]. Smac mimetic (SM) small molecules were initially designed to bind and inhibit XIAP [53,98,103,104,105]. However, these compounds turned out to be primarily active against cIAPs (Figure 2A) [28,99,106,107]. There are two types of Smac mimetics, monovalent and bivalent. The monovalent compounds utilize a single AVPI binding motif to bind IAP proteins, while the bivalent compound has two AVPI binding motifs linked together through a linker. The bivalent Smac mimetics are 100–1000 times more potent than the monovalent Smac mimetic, and the ability of bivalent compounds to bind both BIR2 and BIR3 of XIAP provide better inhibition of XIAP [97,108]. Significantly, both monovalent and bivalent Smac mimetics induce proteasomal degradation of cIAPs but not XIAP [71,109,110]. Degradation of cIAPs by Smac mimetics inhibit the NF-κB canonical pathway by preventing ubiquitylation of RIPK1 by cIAPs. This leads to the formation of a complex containing RIPK1, caspase 8 and FADD, which promotes apoptosis (Figure 2A) [31,32,111,112]. In addition, the depletion of cIAPs by Smac mimetics results in stabilization of NIK (NF-κB inducing kinase) and constitutively activates the non-canonical NF-κB signaling pathway [28,30,31,32,33,34,35]. This results in the expression of NF-kB target genes, such as TNFα which induces the formation complex II which induces apoptosis (Figure 2A) [110,113,114,115,116,117]. However, in cells expressing high levels of RIPK3, RIPK3 is recruited to the RIPK1, caspase-8 and FADD complex to induce necroptosis (Figure 2A) [109,118,119,120,121,122]. Furthermore, in certain cancer cells the absence of XIAP, cIAPs, death receptor stimulation, and treatment with Smac mimetics results in formation of a ripoptosome complex. This ripotosome complex contains FADD, Caspase 8, and RIPK1/3 inducing either apoptosis or necroptosis depending on RIPK3 levels [114,123,124,125,126,127]. In particular, Smac mimetic compounds SM130 and SM114 are selective for degradation of cIAP1 with reduced binding affinity for XIAP [99]. Birinapant (TL32711) is a bivalent compound that displays preferential binding to cIAP1 relative to cIAP2 and XIAP, which is currently being tested in clinical trials [128,129,130,131]. It is a potent IAP-inhibitor and was well-tolerated at doses that sustained target inhibition [91,128,132]. The mechanism by which Smac mimetics (BV6, MV1) induce the degradation of cIAPs is through inducing a conformational change in cIAPs that causes their ubiquitylation and degradation [28,133]. However, most cancer cell lines tested were resistant to the treatment of Smac mimetic [113,134,135]. To overcome the resistance of these cancers to anti-tumorigenic drugs, combination therapies with other anticancer drugs are being explored [136,137,138]. Some studies have reported accelerated disease growth after treatment with the monovalent Smac mimetic LCL161 in a lymphoma mouse model, and a cytokine release syndrome that showed an increased TNFα levels in patients treated with LCL161 [139,140]. These observations raise the important question whether Smac-mimetics exert their effects through apoptosis, inflammation, or necroptosis [122,141,142].

The current small molecule IAP antagonists bind and degrade cIAPs while binding XIAP with lower affinity [99,128,129,130,131]. A major unmet goal of the pharmaceutical industry is therefore, to develop potent and specific small molecules that selectively degrade XIAP [87,143]. To address this need, we generated small-molecule ARTS-mimetics that can bind directly to the unique sequence of ARTS in the BIR3 domain of XIAP, but not to cIAPs. These compounds promote XIAP ubiquitylation and degradation via the UPS (Figure 2B) [79].

We previously showed that small peptides encompassing the binding site of ARTS to XIAP can promote cell death in cancer cells [78,144]. This provides proof-of-concept that mimicking the function of ARTS to antagonize XIAP can promote apoptosis. Next, we performed a structure-based computational screen analyzing 600,000 compounds to identify candidates predicted to bind the unique binding pocket for ARTS in XIAP/BIR3 (performed by BioSolveIt Ltd.). We identified 100 molecules with highest affinity scores of docking to the unique binding site of ARTS in BIR3/XIAP. We then synthesized and tested several compounds for their ability to degrade XIAP and promote apoptosis. The small-molecule ARTS mimetics can degrade XIAP and induce apoptosis, as shown by its ability to promote caspase-3 cleavage and PARP cleavage in A375 melanoma and in T-ALL Jurkat cell lines [79]. Some ARTS-mimetics can directly bind to BIR3/XIAP and promote the degradation of XIAP, but not cIAP1 [79]. Moreover, overexpression of XIAP reduced the effect of ARTS mimetics, suggesting that XIAP is the main target of this ARTS mimetic small molecule [79]. ARTS mimetics decrease XIAP and Bcl-2 levels in Sept4/ARTS-null MEFs, indicating that they act similar to ARTS [79]. Furthermore, both ARTS and Smac proteins serve as substrates of XIAP [87,145]. ARTS mimetics directly bind and degrade XIAP. It is expected that ARTS-mimetics increase levels of XIAP-substrates, such as ARTS and Smac themselves, and thereby amplify the efficacy of ARTS-mimetics for cancer cell killing.

These ARTS mimetics provide the basis for developing a new class of specific XIAP-antagonist, which can potently antagonize XIAP by degrading it. Degrading XIAP, as opposed to allosteric inhibition, should require smaller amounts of drugs to promote tumor killing. Moreover, this may facilitate the development of compounds with reduced systemic load and less unspecific cytotoxic effects [143]. 

In conclusion, IAPs are promising targets for cancer therapy since many types of cancer exhibit high levels of IAPs to evade cell death. Here we compare two main antagonists of IAPs, namely Smac and ARTS, and discuss their distinct properties, mode of action, and function. These data indicate that Smac functions as a more specialized cIAPs antagonist, significantly effecting the TNFα/TRAIL pathway, whereas ARTS functions as a tumor suppressor protein (studied in human patients and Sept4/ARTS KO mice) and acts as a physiological XIAP antagonist. Therefore, Smac-mimetics can be primarily useful for targeting tumors with high levels of cIAPs, whereas ARTS-mimetics are expected to be effective against cancers with high levels of XIAP. 

## Figures and Tables

**Table 1:** Comparison of the two IAP-antagonists Smac and ARTS.

Criteria	ARTS	Smac
Sub-cellular localization	Mitochondrial outer-membrane [47]	Mitochondrial inner membrane space [43]
Requirement for MOMP	Acts upstream of MOMP [69].	Acts downstream of MOMP [43,63]
Translocation/release from mitochondria to the cytosol	Caspase-independent, occurs within minutes after apoptotic stimuli [69]	Caspase-dependent, occurs hours after apoptotic stimuli [63]
Binding to BIR3/XIAP	✓ [78]	✓ [43]
Different binding sites within BIR3/XIAP	BIR3/XIAP (aa 272–292) [78,87]	BIR3/XIAP (aa Leu307, Trp310,Glu314,Trp323, Gly306) [43,56]
Containing different binding sequences to XIAP	Contains a unique C-terminal sequence (AIBM) [78,87]	Contains an IBM (AVPI/F) sequence [43,63,88]
Degradation of XIAP via the ubiquitin proteasome-system	✓ [67]	X [71]
Degradation of cIAPs via the ubiquitin proteasome-system	X [67,77]	✓ [71]
Over-expression phenotype	Sufficient to induce apoptotic cell death in a variety of cultured cell lines [52,69]	Enhances apoptosis in combination with additional apoptotic stimuli [43].
Knockout (KO) mouse phenotype	Sept4/ARTS deficiency promotes spontaneous tumorigenesis. Sept4/ARTS KO mice develop various types of tumors, mainly lymphoma and leukemia [51,85]. MOMP and the release of Cyto c/Smac from mitochondria are delayed in Sept4/ARTS KO cells [69]. Sept4/ARTS KO mice contain elevated XIAP levels [86]. Sept4/ARTS KO mice have increased numbers of stem and progenitor cells, which are resistant to apoptosis [40,41,85,86]. The resistance of Sept4/ARTS-null hematopoietic stem and progenitor cells (HSPCs) to apoptosis and the cell-autonomous lymphoproliferation is suppressed by the loss of XIAP function in Sept4/ARTS/XIAP double-knockout mice [75].	Smac deficiency does not cause spontaneous tumorigenesis [73,74,75]. Knockout mice have no detectable apoptotic defects in vivo [73,74]. Loss of *Smac* in mice led to elevated levels of cIAP1 and cIAP2 and XIAP expression levels remain intact in Smac KO cells [74,75]. Smac-KO cells were resistant to apoptosis induced by NSAIDs and TRAIL [74].
